# Adipokine Signaling Pathways in Osteoarthritis

**DOI:** 10.3389/fbioe.2022.865370

**Published:** 2022-04-19

**Authors:** Chaofan Zhang, Yunzhi Lin, Chun Hoi Yan, Wenming Zhang

**Affiliations:** ^1^ Department of Orthopaedic Surgery, The First Affiliated Hospital of Fujian Medical University, Fuzhou, China; ^2^ Department of Stomatology, The First Affiliated Hospital of Fujian Medical University, Fuzhou, China; ^3^ Department of Orthopaedics & Traumatology, The University of Hong Kong, Pokfulam, Hong Kong SAR, China

**Keywords:** adipokine, osteoarthritis, signaling pathway, cartilage, degeneration, obesity

## Abstract

Osteoarthritis (OA) is a debilitating joint disease that affects millions of individuals. The pathogenesis of OA has not been fully elucidated. Obesity is a well-recognized risk factor for OA. Multiple studies have demonstrated adipokines play a key role in obesity-induced OA. Increasing evidence show that various adipokines may significantly affect the development or clinical course of OA by regulating the pro/anti-inflammatory and anabolic/catabolic balance, matrix remodeling, chondrocyte apoptosis and autophagy, and subchondral bone sclerosis. Several signaling pathways are involved but still have not been systematically investigated. In this article, we review the cellular and molecular mechanisms of adipokines in OA, and highlight the possible signaling pathways. The review suggested adipokines play important roles in obesity-induced OA, and exert downstream function *via* the activation of various signaling pathways. In addition, some pharmaceuticals targeting these pathways have been applied into ongoing clinical trials and showed encouraging results. However, these signaling pathways are complex and converge into a common network with each other. In the future work, more research is warranted to further investigate how this network works. Moreover, more high quality randomised controlled trials are needed in order to investigate the therapeutic effects of pharmaceuticals against these pathways for the treatment of OA. This review may help researchers to better understand the pathogenesis of OA, so as to provide new insight for future clinical practices and translational research.

## 1 Introduction

Osteoarthritis (OA) is the most prevalent arthritis worldwide and a leading cause of pain and physical disability in nearly 10% of males and 18% of females aged 60 years and older (Glyn-Jones et al., 2015). OA is a degenerative disease of the entire joint and occurs most commonly in the hip and knee, characterized by a gradual loss of articular cartilage and remodeling of the subchondral bone (Hochberg et al., 2013). The symptoms and signs of OA include pain, stiffness, deformity and disability, which significantly affect the quality of life of patients (Sharma, 2021). It was estimated that from 1990 to 2019, the number of people affected by OA increased by 48% (Hunter et al., 2020). It should also be noted that the incidence of OA is rising even among young and physically active people (Long et al., 2020).

**FIGURE 1:**
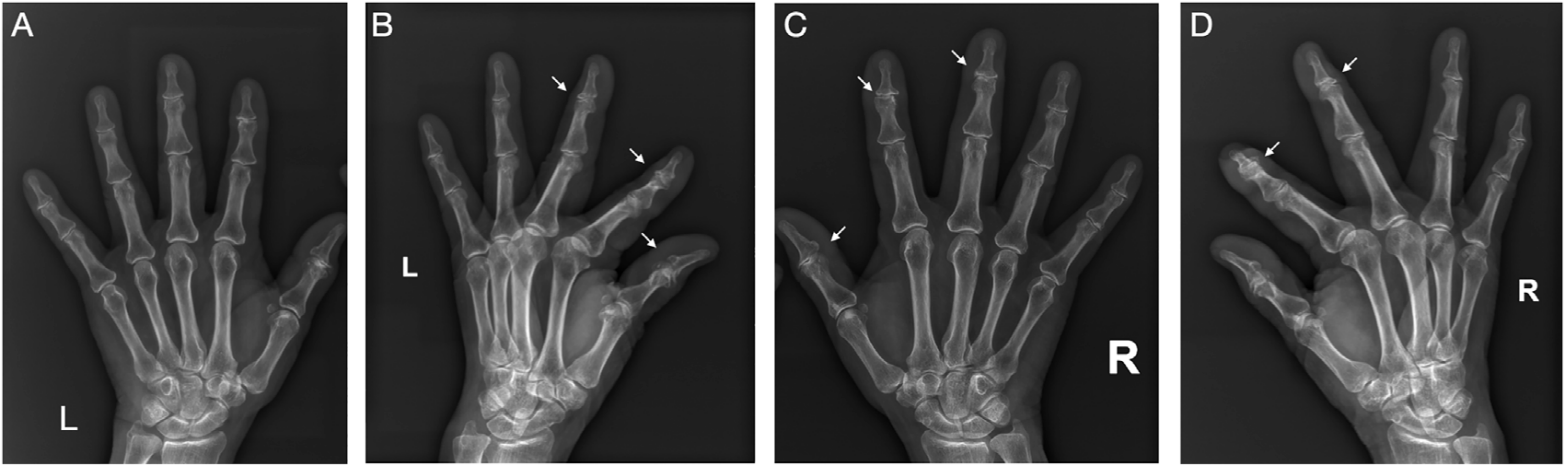
Typical X-ray films of OA in hand. This is the X-ray films of both hands of a 71-year-old obese woman (BMI 28.0). Significant manifestations of OA (narrowing of joint space, osteophyte formation, and subchondral bone sclerosis) can be seen in these non-weight-bearing joints. **(A)** Frontal view of left hand; **(B)** Lateral view of left hand; **(C)** Frontal view of right hand; **(D)** Lateral view of right hand.

The pathogenesis of OA has not yet been fully elucidated. Old age, female sex and obesity are primary risk factors of OA (Collins et al., 2020). Obesity is one of the modifiable risk factors for OA, and was conventionally believed to cause OA *via* the increased mechanical loading on weight-bearing joints, leading to “wear and tear” (Zhang et al., 2018). However, recent evidence showed that this might not be the only pathogenesis. Studies have demonstrated that OA is also common in non-weight bearing joints, such as hand joints, and is more prominent in obese patients, which indicates that OA is not simply a “wear and tear” disease (Neumann et al., 2016; Favero et al., 2022) (Figure 1). Epidemiology study has shown that hand OA was seen in ∼60% of North American and European adults ≥65 years of age—far greater than the ∼33% found in the knee and ∼5% in the hip (Plotz et al., 2021). Increasing evidence suggests that there are multiple subtypes of OA that reflect a complex and multifactorial nature, in which obesity-induced OA has been proposed as a new phenotype of OA that displays a unique characteristic (Sun et al., 2021).

The low-grade chronic inflammation seen in obesity-induced OA might be the main characteristic which are different from other phenotypes of OA (Sun et al., 2021). In obese patients, the cytokines produced and released by adipose tissues, which are also termed as “adipokines,” might play important roles (Conde et al., 2015). In this process, adipose tissue secretes various adipokines (leptin, adiponectin, resistin, visfatin, omentin, vaspin, retinol binding protein 4, etc.) and cytokines [interleukin (IL)-1, IL-6, IL-8, tumor necrosis factor (TNF)-α, etc.], and contribute to the degeneration of chondrocyte and breakdown of extracellular matrix (Urban and Little, 2018; Giardullo et al., 2021). The synovial adipokines might come from the secretion of the infrapatellar fat pad itself or from the blood circulation system permeating the synovial membrane and entering the joint cavity. It’s close association with OA have been widely reported in literature (Collins et al., 2020). Clinical data has shown that there was a positive correlation between synovial adipokine level and OA activity index in elderly women with knee OA (Eldjoudi et al., 2022; Ilia et al., 2022).

Several mechanisms have been proposed. First, studies have shown that the single nucleotide polymorphism rs182052 in the ADIPOQ gene encoding adiponectin may modify individual susceptibility to knee OA (Jiang et al., 2021). In addition, dipokines regulate the metabolic balance of joints by regulating cytokines, chemokines, matrix degrading enzymes, and cell growth/differentiation factors (Xie and Chen, 2019). Many studies have shown that adipokines such as leptin and adiponectin released from the synovium, infrapatellar fat pad and osteophytes can upregulate the levels of inflammatory cytokines such as prostaglandin E2 (PGE2), IL-6, IL-8, vascular cell adhesion molecule (VCAM)-1, and TNF-α in knee synovial fluid, and even infiltrating cartilage and activating the degenerative cascade (Ushiyama et al., 2003; Tang et al., 2007; Tong et al., 2008; Moilanen et al., 2009; Conde et al., 2012; Yang et al., 2013; Tsuchida et al., 2014). During the process, multiple signaling pathways were associated, including AMP-activated protein kinase (AMPK)/mammalian target of rapamycin (mTOR), nuclear factor-κB (NF-κB), mitogen-activated phosphokinase (MAPK), etc.

The detailed pathophysiology by which adipokines lead to the onset and progression of OA is not fully understood. To our knowledge, no review has examined the adipokine signaling pathways in OA. In this context, this review aims to summarize the signaling pathways associated with adipokine in OA. We searched for articles containing the key terms “osteoarthritis,” “obesity,” “adipokine,” and “signaling pathway.” A total of over 200 publications were reviewed. Specifically, the reports on the signaling pathways involving in adipokine-associated OA were carefully reviewed. Publications on other phenotypes of OA, such as traumatic OA, were excluded to avoid selection bias. In addition, a structured search in the clinicaltrials.gov database was performed, and all phase II and phase III trials with pharmaceuticals against signaling pathways for the treatment of OA were selected. The purpose of this review is to help to reveal the underlying biological mechanism, so as to develop new therapeutic targets or biomarkers, and eventually provide insights for the development of preventive strategies and effective treatments.

## 2 Adipokine Signaling Pathways in Osteoarthritis

### 2.1 Main Signaling Pathways

#### 2.1.1 AMP-Activated Protein Kinase/Mammalian Target of Rapamycin Signaling Pathway

AMPK is a highly conserved cell energy metabolism regulator that plays an important role in the regulation of cell growth and survival and energy metabolism in the body (Hardie, 2014; Garcia and Shaw, 2018). mTOR belongs to the phosphatidylinositol kinase-related kinase family. It is an atypical serine/threonine protein kinase that is highly evolutionarily conserved and is mainly involved in regulating cell growth, proliferation, apoptosis, and autophagy (Chen and Zhou, 2020). There are two types of mTOR protein complexes: mTORC1 and mTORC2.

AMPK/mTOR was also found to be associated with the pathogenesis of OA. When the AMPK signaling pathway is activated, the phosphorylation of AMPK blocks the phosphorylation of the mTOR signaling pathway, inhibits the IL-1β-stimulated catabolic response, downregulates the expression of MMPs and phospho-NF-κB, decreases the levels of apoptotic markers, and eventually regulates the progression of OA (Zhou et al., 2017). The AMPK/mTOR signaling pathway is involved in cartilage degeneration and chondrocyte aging (Feng et al., 2020; Zheng et al., 2021). The activation of AMPK promotes autophagy of chondrocytes and inhibits the production of inflammatory cytokines, such as IL-6 and TNF-α, in OA (Zhao et al., 2018).

Adiponectin is the most abundant adipocytokine secreted by adipocytes and a critical member in glucose metabolism and energy balance (Chen et al., 2015). It has strong anti-inflammatory and anti-apoptotic properties, which are closely related to the development and progression of OA. Adiponectin receptors 1 and 2 (AdipoR 1 and AdipoR 2) are two major transmembrane receptors that interact with adiponectin (Harasymowicz et al., 2021). Kang et al. (2010) reported that adiponectin increased the expression of matrix metalloproteinase (MMP)-1, MMP-3, MMP-13, and inducible nitric oxide synthase (iNOS) in human OA chondrocytes through the AdipoR1/2 and AMPK signaling pathways, resulting in the degradation of cartilage matrix. Berendoncks et al. (2013) and Tong et al. (2011) also found that adiponectin may activate AMPK through AdipoR1, which affects the MMP-3 promoter and leads to cartilage destruction. In addition to its effects on chondrocytes, adiponectin can stimulate the proliferation, differentiation and mineralization of osteoblasts in autocrine and/or paracrine fashion through the AMPK signaling pathway (Kanazawa et al., 2007). Chen et al. (2015) further demonstrated that adiponectin promotes osteogenic differentiation of human adipose-derived stem cells by activating the AMPK pathway. Furthermore, adiponectin can also induce intercellular adhesion molecule-1 (ICAM-1) expression *via* AMPK to promote the adhesion of monocytes to human OA synovial fibroblasts (Chen et al., 2014). Moreover, AMPK plays an important role in the expression of vascular cell adhesion molecule-1 (VCAM-1) in human and mouse chondrocytes induced by adiponectin and leptin and makes cartilage degradation permanent (Conde et al., 2012).

Leptin is a ubiquitous fat factor produced by fat and other tissues that regulates food intake and energy consumption. The increased expression of leptin and the enhanced effect of leptin on the infrapatellar fat pad, synovium, articular cartilage and bone are also involved in the pathogenesis of OA (Gao et al., 2020). The role of AMPK/mTOR in leptin-induced OA has not been widely reported. Huang et al. (2016) found that the increase in leptin levels in patients with OA significantly stimulated the expression of lysyl oxidase-like 3. Increased lysyl oxidase-like 3 expression induces chondrocyte apoptosis, activates mTORC1 and inhibits chondrocyte autophagy (Huang et al., 2016).

Resistin induces the expression of proinflammatory factors and chemokines in human cartilage, thus inhibiting the synthesis of cartilage matrix. Epidemiological studies have found that resistin levels in serum and synovial fluid are positively correlated with the severity of OA (Song et al., 2016). The role of AMPK/mTOR in resistin-induced OA is also unknown. Su et al. (2017) studied the mechanism by which low shear stress (2 dyn/cm^2^) regulates the catabolism of resistin in human OA chondrocytes and found that preshear activated the AMPK/sirtuin 1 (SIRT1) signal, but postshear inhibited this signal and regulated the cyclooxygenase-2 expression in human OA chondrocytes induced by resistin.

#### 2.1.2 Nuclear Factor-κB Signaling Pathway

NF-κB is an ubiquitously expressed transcription factor that plays an important role in cell survival, differentiation, proliferation, aging, inflammation, immune response, and apoptosis (Hayden and Ghosh, 2012). Meanwhile, NF-κB has also been proven to be involved in the occurrence and development of OA, including chondrocyte survival and catabolism, as well as synovial inflammation (Roman-Blas and Jimenez, 2006; Rigoglou and Papavassiliou, 2013; Saito and Tanaka, 2017; Choi et al., 2019).

The destruction of cartilage matrix integrity in OA is caused by the increase in chondrocyte catabolism and apoptosis and the decrease in chondrocyte anabolism (Hwang and Kim, 2015; Zheng et al., 2021). Studies have shown that adipokines can induce the expression of matrix-degrading enzymes and/or proinflammatory mediators in chondrocytes through the NF-κB signaling pathway (Choi et al., 2019). Studies have shown that leptin increases the expression of PGE2, IL-6, IL-8, MMP-1, MMP-3, and MMP-13, a disintegrin and metalloproteinase with thrombospondin motifs (ADAMTS)-4, ADAMTS-5, and ADAMTS-9 genes through the NF-κB signaling pathway (Tong et al., 2008; Moilanen et al., 2009; Koskinen et al., 2011; Yaykasli et al., 2015). The increased production of MMPs and ADAMTSs is a marker of the destruction of cartilage homeostasis and the initiator and promoter of OA (Mobasheri et al., 2017). Additionally, adiponectin activates p38 and AMPK through AdipoR1, thereby activating NF-κB on the MMP-3 promoter and leading to cartilage breakdown (Tong et al., 2011). Huang et al. (2010) and Tang et al. (2007) also found that the NF-κB signaling pathway may play a critical role in adiponectin promoting the expression of bone morphogenetic protein (BMP)-2 in osteoblasts and IL-6 in human synovial fibroblasts. Moreover, in one study by Li et al. (2017), resistin was found to combine with toll-like receptor-4 through the p38 MAPK and NF-κB signaling pathways, increasing the expression of chemokine ligand 4 in nucleus pulposus cells and leading to macrophage infiltration. Su et al. (2017) found that human OA chondrocytes exposed to different low shear stress modes have an opposite effect on resistin-induced catabolic cyclooxygenase-2 expression, which is closely related to the NF-κB signaling pathway. That is, preshearing over a short duration inhibits the NF-κB-p65 subunit and cAMP response element binding protein to attenuate the resistin effect. However, postshear for a longer duration enhances the resistin effect by activating only the NF-κB-p65 subunit (Su et al., 2017). Furthermore, resistin can mediate osteoclastogenesis by activating NF-κB (Thommesen et al., 2006). Osteoclastogenesis is also an important pathophysiological change of subchondral bone in OA, as OA is usually associated with subchondral bone sclerosis and remodeling. Another common adipokine, visfatin, was found to induce the expression of MMP-3, MMP-12, MMP-13, IL-6, monocyte chemokine protein 1, and keratinocyte chemokine in osteoblasts and chondrocytes *via* NF-κB (Laiguillon et al., 2014; Yang et al., 2015).

Lipocalin 2, an adipokine initially isolated from neutrophil granules, has been considered a factor that may damage chondrocyte phenotype, cartilage homeostasis, and growth-plate development (Conde et al., 2017). Studies have demonstrated that NF-κB is a key member of lipocalin 2-induced OA (Guo et al., 2014; Conde et al., 2016). A novel adipokine, Nesfatin-1, was also found to stimulate NF-κB pathway (Lee et al., 2021).

#### 2.1.3 Janus Kinase/Signal Transducer and Activator of Transcription Signaling Pathway

The Janus Kinase (JAK)/Signal Transducer and Activator of Transcription (STAT) signaling pathway is the process by which STAT is phosphorylated and dimerized by JAK and then transported to the nucleus through the nuclear membrane to regulate the expression of related genes (Xin et al., 2020). It is an ubiquitously expressed intracellular signal transduction pathway that participates in many important biological processes, including cell proliferation, differentiation, apoptosis, and immune regulation (Rawlings et al., 2004).

Leptin, in one study, has been proven to induce chondrocyte apoptosis and produce MMP-3, MMP-13, reactive oxygen species, iNOS, NO, and VCAM-1 through the JAK/STAT signaling pathway (Otero et al., 2003, 2005; Koskinen et al., 2011; Conde et al., 2012; Zhang et al., 2016). Ben-Eliezer et al. (2007) also demonstrated that the effects of leptin on growth-plate chondrocytes are specifically mediated by STAT 3. Pallu et al. (2010) further found that 500 ng/ml leptin triggers signal transduction through the STAT signaling pathway and thus induces the expression of insulin growth factor (IGF)-1, type 2 collagen, tissue inhibitors of metalloproteinase (TIMP)-2 and MMP-13. Although leptin at 100 ng/ml failed to activate STAT 3, it could induce STAT 1α phosphorylation in the chondrocytes of obese patients (Pallu et al., 2010). Leptin receptor activation initiated by leptin binding was reported to activate multiple signaling pathways including JAK/STAT (Eldjoudi et al., 2022).

Lipocalin 2 is a novel adipokine that has a negative effect on articular cartilage and triggers the catabolism and inflammatory response of chondrocytes in OA. Guo et al. (2014) suggested that lipocalin 2 is a regulator of macrophage polarization and STAT 3 signaling pathway activation. Studies have also shown that IL-1 induces the expression of lipocalin 2 in chondrocytes through JAK2 (Conde et al., 2017).

#### 2.1.4 Mitogen-Activated Phosphokinase Signaling Pathway

MAPK is an important pathway of eukaryotic signal transduction. It regulates many cell biological processes in OA, including proliferation, differentiation, migration and apoptosis (Lan et al., 2021). MAPK signal transduction is carried out *via* a three-level kinase cascade. First, MAPK kinase (MAPKKK) is activated by mitogen-stimulated phosphorylation. On this basis, MAPKKK phosphorylates and activates MAPK kinase (MAPKK). Last, MAPKK phosphorylates MAPK to activate it and then transfers it into the nucleus. MAPK is a proline-directed protein kinase that can phosphorylate serine or threonine residues adjacent to proline. Thus, MAPKs activate many protein kinases, transcription factors, and nuclear proteins, resulting in downstream signal transduction (Cuschieri and Maier, 2005).

Increased MAPK activity has been found in human OA. Currently, p38, c-Jun N-Terminal Kinase (JNK), and Extracellular Signal-Regulated Kinase (ERK)½ in the MAPK family have been investigated and found to be involved in the pathogenesis of OA (Saklatvala, 2007). The p38 and JNK MAPK signaling pathways are important signals involved in the regulation of the inflammatory response. A variety of external stressors, such as cytokines and hypoxia, can cause the phosphorylation of JNK and p38 and lead to the chain reaction of intracellular protein kinase (Shi et al., 2016; Sun et al., 2017). The ERK½ MAPK signal transduction pathway is the key factor determining cell fate when stimulated extracellularly. Its main function is to promote proliferation and regulate cell terminal differentiation (Cuschieri and Maier, 2005).

##### 2.1.4.1 p38 Mitogen-Activated Phosphokinase Signaling Pathway

p38 MAPK plays a pivotal role in cartilage destruction in adipokine associated OA. Researchers have found that leptin dose-dependently stimulates the proliferation of abnormal OA osteoblasts and increases the levels of phosphorylated p38 and ERK½ MAPK (Ben-Eliezer et al., 2007; Mutabaruka et al., 2010). However, inhibition of the p38 MAPK signaling pathway fails to block the effect of leptin on the expression of type X collagen in ATDC5 chondrogenic cells (Ben-Eliezer et al., 2007). Additionally, studies have shown that leptin can increase the expression of PGE2, IL-6, IL-8, nitric oxide synthase (NOS) type II, ADAMTS-4, ADAMTS-5, ADAMTS-9, MMP-1, and MMP-13 in human chondrocytes through the p38 MAPK signaling pathway (Otero et al., 2005, 2007; Moilanen et al., 2009; Koskinen et al., 2011; Yaykasli et al., 2015). Adiponectin, another key adipokine, increases the expression of BMP-2 in osteoblasts, MMP-3 in human chondrocytes, and IL-6 in human synovial fibroblasts *via* the p38 MAPK signaling pathway (Tang et al., 2007; Huang et al., 2010; Tong et al., 2011).

##### 2.1.4.2 c-Jun N-Terminal Kinase Mitogen-Activated Phosphokinase Signaling Pathway

JNK was initially known as a 54-kDa stress-activated protein kinase (SAPK), which is responsible for immune reactions and cellular functions, such as cell growth, differentiation, survival, and apoptosis (Chen et al., 2001; Ge et al., 2017). Many studies have indicated that JNK is phosphorylated and activated in OA and is closely associated with cartilage destruction in the pathogenesis of obesity-induced OA. Wang et al. (2016) demonstrated that dual specificity protein phosphatase 19, a downstream target of leptin, inhibited chondrocyte apoptosis by dephosphorylating JNK. In another study, Lee et al. (2015) found that leptin suppresses TNF-α-induced chondrocyte death through the JNK MAPK signaling pathway in rat articular chondrocytes. Additionally, studies have shown that leptin-induced NO, PGE2, IL-6, and IL-8 production was reduced by inhibitors of JNK (Moilanen et al., 2009). On the other hand, adiponectin leads to the degradation of the OA cartilage matrix and increases the expression of MMPs and iNOS in human OA chondrocytes through the JNK MAPK pathway (Kang et al., 2010). Luo et al. (2005) also found that adiponectin could stimulate human osteoblast proliferation and differentiation through p38 and JNK but not the ERK ½ MAPK signaling pathway. Furthermore, the JNK MAPK signaling pathway is related to adiponectin increasing the expression of ICAM-1 in human OA synovial fluid and promoting the adhesion between monocytes and OA synovial fluid (Chen et al., 2014).

##### 2.1.4.3 Extracellular Signal-Regulated Kinase½ Mitogen-Activated Phosphokinase Signaling Pathway

Although ERK½ MAPK has no direct impact on the degradation of the extracellular matrix like p38 MAPK and JNK MAPK, it mainly mediates the proliferation and hypertrophic differentiation of chondrocytes, leading to cartilage calcification and osteophyte formation (Lin et al., 2021). As the only cell of articular cartilage, the fate of chondrocytes determines the formation and development of adipokine-associated OA.

Leptin, as a proinflammatory fat factor, was found to play a key role in cartilage metabolism by inducing the upregulation of MMP1 and MMP13 expression with concomitant activation of the STAT, MAPK (p38, JNK, and ERK), Akt, and NF-κB signaling pathways. Otero et al. (2005), Otero et al. (2007) strongly suggested that ERK½ MAPK and p38 MAPK play pivotal roles in the activation of leptin-mediated NOS type II. Moreover, ERK has also been reported to be involved in activating lipocalin 2 by IL-1α during OA progression (Conde et al., 2017). Collagen II expression was also found to be stimulated by leptin-medicated MAPK/ERK signaling pathway in an *in vivo* study (Wang et al., 2022).

Chemerin is a novel adipokine identified in 2007. It is highly expressed in the synovial fluid and synovial membrane of patients with OA and is positively correlated with the severity of OA (Huang et al., 2012; Ma et al., 2015). Berg et al. (2010) suggested that the stimulation of chondrocytes with chemerin could lead to the phosphorylation of ERK½ MAPK and Atk. Additionally, the levels of proinflammatory cytokines, such as IL-1β, IL-6, IL-8, TNF-α, MMP-1, MMP-2, MMP-3, MMP-8, and MMP-13, are significantly increased (Berg et al., 2010).

#### 2.1.5 Activating Protein-1 (AP-1) Signaling Pathway

Activating Protein-1 (AP-1) is most often defined as a collective term, which refers to dimeric transcription factors composed of Jun (v-Jun, c-Jun, JunB, and JunD), Fos (v-Fos, c-Fos, FosB, Fral-1, and Fra2), activating transcription factor (ATF-2, ATF-3/LRF1, ATF-4, ATF-5, ATF-6B, ATF-7, BATF, BATF-2, BATF-3, and JDP2), and MAF (c-MAF, MAFA, MAFA-B, MAFA-F, MAFA-G, MAFA-K, and Nrl) subunits, which bind to a common DNA site (Bejjani et al., 2019).

The AP-1 signaling pathway may be the signaling pathway of leptin-induced IL-6 production in OA synovial fibroblasts. Yang et al. (2013) found that leptin activated IRS-1/PI3K/Akt signaling and increased the production of IL-6 by binding with the OBRI receptor, thus enhancing the transcriptional activity of AP-1 and eventually leading to the transcription of IL-6. There are three known cis-regulatory elements in the promoter region of the IL-6 gene, including AP-1, NF-κB, and C/EBP-β. Yang et al. (2013) confirmed that leptin-stimulated luciferase activity was eliminated by AP-1 binding site mutation, and an AP-1 inhibitor (curcumin) and c-jun siRNA could antagonize the increase in IL-6 expression mediated by leptin. In addition, the stimulation of OA synovial fibroblasts by leptin increased c-Jun phosphorylation in a time-dependent manner. Moreover, leptin increased the binding of c-Jun to the AP-1 (-312 to -39) element in the IL-6 promoter (Yang et al., 2013).

AP-1 is a critical transcription factor that controls ICAM-1 production and cell movement during the progression of adiponectin-induced OA. By exploring the effect of adiponectin on the expression of ICAM-1 in synovial fibroblasts, researchers found that adiponectin led to the transactivation of ICAM-1 expression by enhancing the binding of the AP-1 transcription factor and ICAM-1 promoter and promoted the adhesion of monocytes to human OA synovial fibroblasts through the AP-1 pathway (Chen et al., 2014).

#### 2.1.6 Insulin Receptor Substrate-1 Signaling Pathway

Insulin Receptor Substrate-1 (IRS-1) is the main substrate of receptor tyrosine kinase for insulin and insulin-like growth factor 1, and it is also a substrate of IL-4-activated tyrosine kinase and has been associated with adipokine associated OA pathophysiology (Myers et al., 1994). Specifically, leptin in the synovium may play a proinflammatory role in the pathogenesis of OA through the IRS-1 signaling pathway (Gao et al., 2020). Studies have shown that leptin induces the production of IL-8 in OA synovial fibroblasts *via* an IRS-1/PI3K/Akt/NF-κB-dependent pathway (Tong et al., 2008). Yang et al. and Tang et al. found that leptin also increased the production of IL-6 *via* the activation of the IRS-1/PI3K/Akt/NF-κB signaling pathway (Yang et al., 2013).

#### 2.1.7 Phosphatidyl Inositol 3 Kinase-Protein Kinase B Signaling Pathway

The Phosphatidyl Inositol 3 Kinase (PI3K)-Protein Kinase B (Akt) signaling pathway functions in many cellular processes that are essential for homeostasis, including the cell cycle, survival, metabolism, inflammation, and apoptosis (Tang et al., 2018). Molecules, such as adipokines and cytokines, can activate receptor tyrosine kinases and G protein-coupled receptors and then activate PI3K to generate phospholipase (De Santis et al., 2019). Akt, as a downstream effector of PI3K, is subsequently activated by these signals. Activated Akt is transferred to other cellular compartments to activate various downstream substrates, including metabolic enzymes, protein kinases, small G protein regulators, E3 ubiquitin ligases, and transcription factors (Manning and Toker, 2017). The PI3K-Akt signaling pathway plays an important regulatory role in cartilage homeostasis, subchondral bone dysfunction and synovitis (Sun et al., 2020). A previous study suggested that the PI3K-Akt signaling pathway is downregulated in human cartilage with OA compared with normal cartilage (Rosa et al., 2011). Specifically, inhibition of the PI3K-Akt signaling pathway can promote the proliferation, apoptosis, and autophagy of articular chondrocytes and reduce the inflammatory response in OA (Xue et al., 2017; Sun et al., 2020). Many studies have shown that adipokines can regulate the progression of OA through the PI3K-Akt signaling pathway.

Visfatin is also a newly discovered proinflammatory adipokine produced by visceral white adipose tissue, which can be found in muscle, bone, synovium, and cartilage (Franco-Trepat et al., 2019). Vascular endothelial growth factor and endothelial progenitor cells are critical factors that promote angiogenesis of the pannus in OA (Kiewisz et al., 2016; Patel et al., 2016; MacDonald et al., 2018). Studies have shown that visfatin inhibits the synthesis of miRNA-485-5p through the PI3K-Akt signaling pathway, which affects the expression of vascular endothelial growth factor in OA synovial fluid and the angiogenesis of endothelial progenitor cells (Tsai et al., 2020).

Vaspin (visceral adipose tissue-derived serine protease inhibitor), a novel adipokine, is produced by skeletal muscle and participates in bone metabolism in an obesity-dependent manner (Tarabeih et al., 2020). Researchers have declared that vaspin can inhibit osteogenic differentiation by activating the PI3K-Akt signaling pathway. During this process, PI3K-Akt and miRNA-34c constitute a modulation loop and control the expression of each other (Liu et al., 2016). Another study has also indicated that PI3K-Akt is critical in Vaspin-induced proliferation of BMSc in OA (Wang et al., 2021).

Apelin is an adipokine involved in the pathogenesis and angiogenesis of OA (Wang et al., 2020). It plays a catabolic role in cartilage metabolism. Specifically, apelin could stimulate chondrocyte proliferation and significantly increase the catabolic factors MMP-1, MMP-3, MMP-9, ADAMTS-4, ADAMTS-5, and the proinflammatory cytokine IL-1β as well as reduce the level of type II collagen (Hu et al., 2010). Chang et al. first proved that apelin stimulates IL-1β expression by activating the PI3K and ERK signaling pathways and inhibiting the downstream expression of miRNA-144-3p in OA synovial fibroblasts (Chang et al., 2020).

Omentin-1, also known as intelectin-1, is a newly identified anti-inflammatory adipokine involved in lipid metabolism (Chai et al., 2020). It has been reported that omentin-1 can stimulate the proliferation and inhibit the differentiation of mouse osteoblasts (Xie et al., 2011, 2012). Wu et al. (2013) found that omentin-1 could induce human osteoblast proliferation through the PI3K-Akt signaling pathway.

Furthermore, stimulation of OA synovial fluid with leptin leads to time-dependent phosphorylation of Akt (Yang et al., 2013). Additionally, leptin can promote the expression of IL-6 in human OA synovial fluid and IL-8 in human synovial fibroblasts through the PI3K-Akt signaling pathway (Tong et al., 2008; Yang et al., 2013). In addition, PI3K was demonstrated to be involved in leptin and adiponectin increasing the expression of VCAM-1 in human and mouse chondrocytes (Conde et al., 2012). Moreover, PI3K could induce the expression of lipocalin 2 in cartilage and affect cartilage homeostasis (Guo et al., 2014). Chemerin can also induce Akt phosphorylation in chondrocytes and increase the levels of proinflammatory cytokines, including IL-6, IL-8, IL-1β, TNF-α, and MMPs (Berg et al., 2010).

#### 2.1.8 Peroxisome Proliferator-Activated Receptor Signaling Pathway

Peroxisome Proliferator-Activated Receptor (PPAR) is a ligand-activated transcription factor that plays a key regulatory role in lipid metabolism and energy homeostasis (Huang et al., 2021). There are three PPAR isotypes, namely, α, γ and β/δ (Dubois et al., 2017).

PPARs regulate the homeostasis of articular cartilage *via* various pathways and reduce the inflammatory response of OA cartilage. First, PPAR-γ, known as the main adipogenesis regulator, may affect the deposition of fat in skeletal muscle and connective tissue. Fat deposition is an important risk factor for knee OA. The main adipose tissue of the knee joint is the infrapatellar fat pad, which can produce inflammatory cytokines and adipokines. Therefore, the activation of the PPAR-γ signaling pathway may promote adipogenesis, which could be related to the pathological changes in the infrapatellar fat pad in OA (Cordani et al., 2013; Reggio et al., 2019, 2020; Cerquone Perpetuini et al., 2020). Meanwhile, the lack of PPAR-γ in articular cartilage may accelerate the cartilage destruction and progression of OA by reducing chondrocyte differentiation and proliferation and increasing catabolic activity (Monemdjou et al., 2012; Vasheghani et al., 2013, 2015). Studies have shown that stimulation of adiponectin leads to increased PPAR-α ligand activity, fatty acid oxidation and glucose uptake in skeletal muscle (Kanazawa et al., 2007). It was also reported that the PPAR-γ coactivator (PGC)-1α critically mediates anti-catabolic activity in human knee OA chondrocytes (Wang et al., 2015). In addition, the activation of PPAR was found to significantly reduce the synthesis of key adipokine-associated OA mediators, such as MMPs, ADAMTS-5, TNF-α, PGE2, IL1-β, IL-6 and nitric oxide (NO), and inhibit the activation of the ERK½ MAPK, p38, AP-1, and NF-κB signaling pathways (Boileau et al., 2007; Clockaerts et al., 2011; Fahmi et al., 2011; Scirpo et al., 2015). Ratneswaran et al. (2015) also reported that the activation of PPAR-δ could induce the upregulation of proteolytic active genes in cartilage principal extracellular matrix as well as increase the degradation of aggrecan and the release of glycosaminoglycan in knee joint explants. Cartilage-specific PPAR-δ knockout mice showed a strong protective effect on cartilage degeneration in the destabilization of the medial meniscus model of post-traumatic OA (Ratneswaran et al., 2015). Moreover, activated PPAR can inhibit the activation of NF-κB induced by adipokines or promote its inactivation through the following mechanisms, leading to the inhibition of inflammation. PPAR interferes with the activation of NF-κB in the inflammatory response mainly by upregulating the expression of IκBα, sirtuin 1, and phosphatase and tensin homolog (Korbecki et al., 2019). A possible mechanism of PPAR promoting NF-κB inactivation is that PPAR, as an E3 ubiquitin ligase, can physically interact with p65 NF-κB, which leads to inactivation and ubiquitination of p65 NF-κB and finally results in the proteolytic degradation of p65 NF-κB (Hou et al., 2012). Furthermore, adiponectin could selectively activate human monocytes into anti-inflammatory M2 macrophages through the PPAR-α/γ signaling pathway, thus controlling the progression of OA (Lovren et al., 2010).

#### 2.1.9 Wnt/β-Catenin Signaling Pathway

Wnt is an extracellularly secreted glycoprotein. Its signal transduction involves 19 Wnt genes and various Wnt receptors that regulate the canonical β-catenin-dependent signaling pathway (Wang et al., 2019). The overactivation of the Wnt/β-catenin signaling pathway is related to the degradation process of OA (Huang et al., 2020; Palma and Nalesso, 2021). Wang et al. (2017) investigated the effects of adiponectin on osteogenic differentiation and bone formation of bone mesenchymal stem cells (BMSCs). They conducted both *in vivo* and *in vitro* studies and found higher gene and protein expression levels of osteogenesis-related genes and Wnt/β-catenin pathway-related factors β-catenin and cyclin D1 in adiponectin transgenic BMSCs and rats. Therefore, adiponectin can facilitate osteogenic differentiation and bone formation of BMSCs *via* the Wnt/β-catenin signaling pathway (Wang et al., 2017).

### 2.2 Other Pathways

#### 2.2.1 RhoA/ROCK Signaling Pathway

Rho kinase (ROCK), a serine/threonine kinase, is a downstream effector of member A of the Ras homologous gene family (RhoA) and is involved in regulating cell migration, proliferation and survival (Cai et al., 2021). The abnormal activation of RhoA/ROCK signaling is involved in the early response to abnormal mechanical stimulation, which is considered to be a promoter of the progression of OA. RhoA/ROCK interacts with OA pathological factors, including β-catenin, transforming growth factor (TGF), epidermal growth factor receptor (EGFR), IL-1, insulin-like growth factor-1 (IGF-1) and leptin, and induces cartilage degeneration through the degradation of chondrocyte extracellular matrix (Deng et al., 2019). Liang et al. (2011) proved that leptin mediates cartilage cytoskeleton remodeling through the RhoA/ROCK signaling pathway and its downstream mediators LIMK1 and cofilin-2.

#### 2.2.2 Sirtuin-1 Signaling Pathway

SIRT-1 is a histone deacetylase. SIRT-1 regulates gene expression and protein function by deacetylating lysine residues in histones and nonhistones (Matsuzaki et al., 2014). It is reported that SIRT-1 regulates aging and age-related diseases in simple eukaryotes (Chen et al., 2020).

It is widely recognized that SIRT-1 activity plays an anti-catabolic role in chondrocytes in the development of OA (Gabay et al., 2013; Matsuzaki et al., 2014; Goldring and Berenbaum, 2015). Researchers have found that SIRT-1 is involved in the pathogenesis of OA by regulating chondrocyte gene expression and hypertrophy (Fujita et al., 2011). SIRT-1 increases the expression of the gene encoding cartilage extracellular matrix in human chondrocytes and improves the survival rate of human OA chondrocytes by inhibiting apoptosis (Takayama et al., 2009; Gagarina et al., 2010; Goldring and Berenbaum, 2015). Moreover, SIRT-1 could also exert anti-inflammatory effects in different tissues by inhibiting the transcription of pro-inflammatory genes (Michan and Sinclair, 2007). Studies have shown that SIRT-1 inhibits the expression of cartilage-degrading enzymes induced by IL-1β and TNF-α by regulating the NF-κB pathway (Matsushita et al., 2013; Moon et al., 2013). In particular, Su et al. (2017) reported that low shear stress regulates the expression of resistin-induced catabolic cyclooxygenase-2 in human OA chondrocytes *via* the AMPK/SIRT-1 signaling pathway and then NF-κB and cAMP response element binding protein transcription factors. Specifically, AMPK activity and SIRT-1 expression in human OA chondrocytes decrease under resistin-induced cyclooxygenase-2 expression. In addition, the attenuation of the preshear effect and the enhancement of the postshear effect of resistin-induced cyclooxygenase-2 expression are caused by the regulation of AMPK and then SIRT-1 signaling (Su et al., 2017). Furthermore, Patel et al. suggested that omentin-1 reduced the expression of IL-1β-induced senescent factors (including caveolin-1, p21 and PAI-1) and p53 acetylation by ameliorating SIRT1 reduction (Chai et al., 2020).

#### 2.2.3 p53/p21 Signaling Pathway

The p53/p21 signaling pathway is implicated in the progression and severity of OA (Xu et al., 2020). In knee OA cases, the protein and mRNA expression of p53 is significantly higher than that in healthy controls (Zhu et al., 2016). Xu et al. (2020) found that chondrocyte apoptosis and p53 increased during the progression of OA, while the expression of SIRT1 decreased in human cartilage. The deletion of SIRT1 in cartilage leads to the acceleration of OA through the abnormal activation of senescence-associated secretory phenotype, hypertrophy, and apoptosis mediated by p53/p21 (Xu et al., 2020). Zhao et al. (2016) further confirmed that high-dose leptin induces cell cycle arrest and senescence in chondrogenic progenitor cells by activating the p53/p21 signaling pathway and inhibiting the SIRT1 signaling pathway.

#### 2.2.4 Calcium Calmodulin-Dependent Kinase II Signaling Pathway

Calcium Calmodulin-Dependent Kinase II (CaMKII) is a serine/threonine protein kinase and a general integrator of Ca^2+^ signaling (Wei et al., 2018). The upregulation of CaMKII is highly correlated with the pathogenesis and progression of OA and the reactivation of articular cartilage hypertrophy (Saitta et al., 2019; Cai et al., 2021). Studies have demonstrated that activated phosphorylated-CaMKII may play a key role in chondrocyte apoptosis through the MAPK and Akt/mTOR signaling pathways (Wei et al., 2018). Researchers have proven that adiponectin can promote ICAM-1 expression and monocyte adhesion in synovial fibroblasts through the CaMKII signaling pathway (Chen et al., 2014).

#### 2.2.5 Protein Kinase C and Protein Kinase A Signaling Pathways

Many studies have indicated that Protein Kinase C (PKC) and Protein Kinase A (PKA) have a close relationship with adipokine-induced OA. Thommesen et al. revealed that resistin stimulates the proliferation of preosteoblasts (MC3T3-E1) and the differentiation of preosteoclasts (RAW 264.7) through the PKC and PKA signaling pathways (Thommesen et al., 2006). Studies have also shown that vaspin inhibits TNF-α-induced ICAM-1 expression *via* activation of PKC, thereby inhibiting the inflammatory state of vascular smooth muscle cells during OA progression (Phalitakul et al., 2011). In addition, PKC is involved in leptin-induced MMP production (Koskinen et al., 2011).

#### 2.2.6 ATF4/RANKL Signaling Pathway

ATF4 is an osteoblast-specific member of the cyclic AMP response-binding protein (CREB) family. It has been reported that leptin regulates osteoclast differentiation and osteoblast proliferation by using ATF4 and its target gene RANKL as transcription medium through sympathetic signals (Fu et al., 2005). In addition, adipokine-induced IL-6 also triggers osteoclast formation and bone resorption through the ATF4/RANKL signaling pathway (Xie and Chen, 2019).

#### 2.2.7 Bone Morphogenetic Protein-2 Signaling Pathway

BMP-2 is an important growth factor promoting osteogenesis and plays a pivotal role in osteoblastic differentiation and bone formation. It has been found to be expressed at low levels in the synovial fluid of OA patients (Yang et al., 2018). Studies have shown that adiponectin affects the progression of OA by enhancing the expression level of BMP-2 in osteoblasts (Huang et al., 2010).

#### 2.2.8 CCAAT/Enhancer-Binding Protein-β Signaling Pathway

The CCAAT/Enhancer-Binding Protein (C/EBP-β) pathway is a key signaling pathway that regulates the downstream target genes of osteoblastogenesis. The expression of C/EBP-β is a reliable output for measuring the activity of osteoblastogenesis at the cellular level (Wang et al., 2018). It has been confirmed that resistin can activate the C/EBP-β signaling pathway and thus upregulate proinflammatory cytokines and chemokines (Zhao et al., 2019).

#### 2.2.9 Notch Signaling Pathway

Notch is a single-pass transmembrane cell surface receptor that plays a key role in cell fate by regulating cell differentiation and apoptosis. The direct transcription targets downstream of Notch are Hes1, Hes5, Hes7, Hey1, Hey2, and HeyL, of which only Hes1 is highly expressed in articular chondrocytes (Hosaka et al., 2013; Sugita et al., 2015). Activation of the Notch signaling pathway affects the occurrence of OA by enhancing the production of inflammation-related molecules in OA synovial cells and chondrocytes (Saito and Tanaka, 2017). Leptin can stimulate the expression of IL-6 in serum and OA synovial fluid. The upregulated IL-6 then reduces the synthesis of bone proteoglycans and increases the expression of the decomposition factor MMP13 through the Notch signaling pathway, resulting in the degradation of proteoglycans and the suppression of cartilage formation (Zanotti and Canalis, 2013; Yan et al., 2018).

## 3 Signaling Pathways of Osteoarthritis as Diagnostic Biomarker and Therapeutic Targets

Signaling pathway as a diagnostic biomarker of OA is rarely reported in the literature. Most studies on diagnostic biomarkers still focus on collagenous markers of cartilage decomposition, metabolic mediators (mainly adipokines) and inflammatory mediators (mainly cytokines) (Kumavat et al., 2021). Only a few literatures have reported the potential of signaling pathway as a diagnostic biomarker of OA, mainly by means of high throughout sequencing and bioinformatics analysis (Zhang et al., 2020). Therefore, in the future work, whether the signal pathway or its key components can be used for early diagnosis of OA needs further research.

To date, the key management strategies for OA have included non-pharmacological (e.g., education and self-management, exercises, weight loss if overweight), pharmacological (e.g., NSAIDs and intra-articular injection of corticosteroids), and surgical approaches. (Sun et al., 2020). There are currently no approved disease-modifying treatments for osteoarthritis. Although many preclinical studies have reported various signaling pathways involved in OA, as reviewed above, there are still few drugs applied in clinical trials. (Table 1) Moreover, some studies have even been terminated. Our review can provide ideas and directions for more clinical trials in the future.

**TABLE 1:** Ongoing clinical trials investigating inhibitors of signaling pathways as treatment for OA.

Intervention	Mechanism	Targets	Joint	Phase	NCT No.	Recruitment Status
Lorecivivint (SM04690)	Wnt signaling pathway inhibitor	Wnt signaling pathway	knee	Phase 3	NCT04520607	Active, not recruiting
Lorecivivint (SM04690)	Wnt signaling pathway inhibitor	Wnt signaling pathway	knee	Phase 3	NCT04385303	Completed
Lorecivivint (SM04690)	Wnt signaling pathway inhibitor	Wnt signaling pathway	knee	Phase 3	NCT03928184	Completed
Lorecivivint (SM04690)	Wnt signaling pathway inhibitor	Wnt signaling pathway	knee	Phase 2	NCT03706521	Terminated
Lorecivivint (SM04690)	Wnt signaling pathway inhibitor	Wnt signaling pathway	knee	Phase 2	NCT03122860	Completed
Lorecivivint (SM04690)	Wnt signaling pathway inhibitor	Wnt signaling pathway	Not given	Phase 2	NCT02536833	Completed
SAR-113945	I-kappa B kinase inhibitors	NF-κb	knee	Phase 2	NCT01598415	Completed
SAR-113945	I-kappa B kinase inhibitors	NF-κb	Not given	Phase 1	NCT01113333	Completed
SAR-113945	I-kappa B kinase inhibitors	NF-κb	knee	Phase 1	NCT01463488	Completed
SAR-113945	I-kappa B kinase inhibitors	NF-κb	knee	Phase 1	NCT01511549	Completed
PH-797804	p38 MAPK inhibitor	p38 MAPK	Not given	Phase 2	NCT01102660	Completed

## 4 Summary and Future Perspectives

In this review, we summarize the possible adipokine signalling pathways in OA reported in the current literature, including the ones that are common and classic, such as AMPK/mTOR and NF-κB, and some novel pathways, such as C/EBP-β and ATF4/RANKL. Various studies have suggested adipokines play important roles in obesity-induced OA, and exert downstream function *via* the activation of these signaling pathways. In addition, based on these findings, some pharmaceuticals targeting these pathways (such as Wnt, NF-κB, and p38 MAPK) have been applied into ongoing clinical trials and showed encouraging results. Inhibitors or antibodies against some novel pathways have demonstrated excellent results in alleviating OA in preclinical studies. These data might provide novel therapeutic strategies for the treatment of OA, especially obesity-induced OA.

**FIGURE 2:**
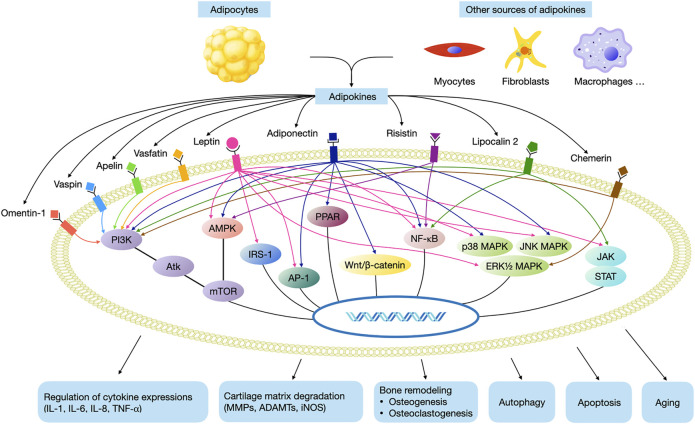
Main adipokine signaling pathways in OA. (AMPK, AMP-activated protein kinase; mTOR, mammalian target of rapamycin; NF-κB, nuclear factor-κB, JAK, janus kinases; STAT, signal transducer and activator of transcription, MAPK, mitogen activated phosphokinase; AP-1, activating protein-1; IRS-1, insulin receptor substrate-1; PI3K, phosphatidyl inositol 3 kinase; Akt, protein kinase B; PPAR, peroxisome proliferator-activated receptor).

However, these signaling pathways are complex and converge into a common network with each other (Figure 2). It has to be noted that none of the adipokines mediates the downstream reaction *via* a single signaling pathway, and a specific signaling pathway is also the common target of several different adipokines. Therefore, in the future work, more research is warranted to further investigate how this network works. Moreover, more high quality randomised controlled trials are needed in order to investigate the therapeutic effects of pharmaceuticals against these pathways for the treatment of OA, as these drugs may serve as disease-modifying drugs. Our review may help researchers to better understand the pathogenesis of OA, so as to provide new insight for future clinical practices and translational research.
